# Associations of Plasma Lipids with Traumatic Brain Injury Outcomes: A Transforming Research and Clinical Knowledge in Traumatic Brain Injury Study

**DOI:** 10.1177/2689288X251395462

**Published:** 2025-11-11

**Authors:** Andrea L.C. Schneider, Benjamin L. Brett, Sabrina Abbruzzese, Danielle K. Sandsmark, Sonia Jain, Xiaoying Sun, Raquel C. Gardner, Geoffrey T. Manley, Lindsay D. Nelson, Ramon Diaz-Arrastia, The TRACK-TBI Study Investigators

**Affiliations:** ^1^ Department of Neurology, University of Pennsylvania Perelman School of Medicine, Philadelphia, Pennsylvania, USA.; ^2^ Department of Biostatistics, Epidemiology, and Informatics, University of Pennsylvania Perelman School of Medicine, Philadelphia, Pennsylvania, USA.; ^3^ Departments of Neurosurgery and Neurology, Medical College of Wisconsin, Milwaukee, Wisconsin, USA.; ^4^ Biostatistics Research Center, Herbert Wertheim School of Public Health and Human Longevity Science at the University of California San Diego, San Francisco, California, USA.; ^5^ Sagol Neuroscience Center, Sheba Medical Center, Ramat Gan, Israel.; ^6^ Department of Neurosurgery, University of California San Francisco, San Francisco, California, USA.

**Keywords:** cholesterol, lipids, outcomes, TBI

## Abstract

Hypocholesterolemia has been observed in acute trauma, but less is known about lipid levels in acute traumatic brain injury (TBI). Our objectives were to (1) compare day 1 post-injury lipid levels from individuals with acute TBI to orthopedic trauma and healthy controls and (2) investigate associations of day 1 post-TBI lipid levels with clinical outcomes over the first-year post-injury. A subset of 266 participants with TBI, 68 orthopedic trauma controls, and 58 healthy controls from the Transforming Research and Clinical Knowledge in TBI Study were included in analyses. Plasma total cholesterol and triglycerides were measured using the enzymatic colorimetric method, and high-density lipoprotein (HDL) was measured via direct homogeneous/enzymatic colorimetric methods. Low-density lipoprotein (LDL) values were calculated using the Friedewald formula. The primary TBI outcome was the Glasgow Outcome Scale–Extended (dichotomized as favorable [score = 5–8] versus unfavorable [score = 1–4] outcome and complete [score = 8] versus incomplete [score = 1–7] recovery; administered at 2 weeks, 3 months, 6 months, and 1-year post-injury). Age- and sex-adjusted total cholesterol and LDL cholesterol were lower among participants with TBI compared with orthopedic trauma and healthy controls (total cholesterol: 116.7 mg/dL [95% confidence interval or CI = 112.1–121.3] versus 137.8 mg/dL [95% CI = 129.1–146.5] and 130.2 mg/dL [95% CI = 120.6–139.9]; LDL cholesterol: 59.3 mg/dL [95% CI = 55.8–62.9] versus 77.7 mg/dL [95% CI = 71.1–84.3] and 72.4 mg/dL [95% CI = 65.1–79.7]). Adjusted levels of HDL cholesterol and triglycerides were similar by group. Among participants with TBI, higher day 1 post-injury total cholesterol, LDL, and triglyceride levels were associated with greater adjusted odds of favorable versus unfavorable outcome after injury. In conclusion, our study found that day 1 post-injury levels of total and LDL cholesterol were lower among individuals with TBI compared with both orthopedic trauma and healthy controls. Furthermore, our results suggest that individuals who did not have acutely low total cholesterol and LDL cholesterol, and, to a lesser extent, triglycerides, had better functional outcomes over the first-year post-TBI.

## Introduction

Traumatic brain injury (TBI) is common and associated with significant long-term disability.^1–4^ Identifying measurable factors in the acute post-injury period that are associated with long-term clinical outcomes is needed to inform clinical prognostication. In the broader trauma literature, hypocholesterolemia has been observed in critically ill injured individuals, and lower acutely measured lipid levels have been associated with increased mortality risk in these populations.^5–7^ Decreased synthesis of cholesterol precursors, inflammation-induced degradation of lipid particles, and increases in capillary permeability are potential mechanisms hypothesized to underlie the hypocholesterolemia observed in those with severe polytrauma and critical illness; similar pathophysiology may exist in TBI.^8,9^ Less is known about lipid levels in the acute TBI setting, though several smaller studies (sample size <200) have suggested that hypocholesterolemia in the acute injury setting is also observed in TBI and that lower lipid levels in acute TBI are associated with higher inflammatory cytokines,^10^ diffuse axonal injury,^11^ disruption of white matter microstructural integrity,^12^ and greater risk for hemorrhage progression.^13^ There remains a need to better understand lipid values in the acute TBI setting (across injury severities) and their associations with long-term clinical outcomes.

Leveraging data from the Transforming Research and Clinical Knowledge in TBI (TRACK-TBI) Study, our objectives were to (1) compare day 1 post-injury plasma lipid levels from participants with TBI to both orthopedic trauma controls and healthy controls and (2) investigate associations of day 1 post-injury lipid levels with outcomes over the first-year post-injury among participants with TBI. We hypothesized that participants with TBI would have similar lipid levels to orthopedic trauma controls and lower lipid levels compared with healthy controls. We further hypothesized that participants with TBI who did not have acutely low day 1 post-injury lipid levels would have better outcomes over the first-year post-injury, independent of injury severity.

## Methods

### Study design and participants

The TRACK-TBI Study is a prospective cohort of individuals with acute non-penetrating TBI events who presented to one of 18 Level 1 trauma centers in the United States and underwent a clinically indicated head computed tomography (CT) exam within 24 h of injury (TBI defined in accordance with the American Congress of Rehabilitation Medicine definition).^14,15^ The study also enrolled orthopedic trauma controls and healthy controls. Orthopedic trauma controls are individuals who presented for orthopedic injuries and showed no evidence of signs or symptoms of head trauma. Healthy controls are individuals without a history of TBI within the past year who were recruited from the friends and family of enrolled TBI patients. Eligible individuals were enrolled between February 26, 2014, and August 8, 2018. Individuals with TBI (and orthopedic trauma controls) participated in follow-up visits at 2 weeks (in-person), 3 months (telephone), 6 months (in-person), and 1 year (in-person) post-injury; healthy controls participated in one in-person visit.^14^

The TRACK-TBI Study was approved by institutional review boards (IRBs) at each study site (Baylor College of Medicine, Massachusetts General Hospital/Spaulding Rehabilitation Hospital, University of California, San Francisco, University of Cincinnati, University of Maryland, University of Miami, University of Pittsburgh, University of Texas at Austin, University of Texas Southwestern, University of Washington, Virginia Commonwealth University, University of Pennsylvania, Emory University, Medical College of Wisconsin, University of Utah, Indiana University, Hennepin Healthcare, University of Colorado). The overall study received approval from the IRB of record (i.e., Ethics Reviewer) at the University of California, San Francisco (Protocol Number: 10-00111). Each participant (or their legally authorized representative) provided written informed consent.

Of the TRACK-TBI participants aged ≥17 years with TBI, a Glasgow Coma Scale (GCS) score-stratified (GCS 13–15; GCS 3–12) random subset with available day 1 post-injury stored frozen plasma and 6 months post-injury Glasgow Outcome Scale–Extended (GOSE) TBI Version^16^ data was eligible for inclusion in the present analyses. Of these 278 participants with TBI, 5 were excluded for missing lipid data, and 7 were excluded for missing covariates included in statistical models, leaving 266 participants with lipid and GOSE data. Among these 266 participants with TBI, 170 also had outcome data on injury-related symptoms, psychological distress, and satisfaction with life, and 154 also had cognitive outcome data. Day 1/enrollment lipid data were also measured among 68 orthopedic trauma controls and 58 healthy controls.

### Lipids

Blood samples were obtained from participants within 24 h of injury (TBI and orthopedic injury controls) and on the day of enrollment (healthy controls). Blood samples were acquired, processed, aliquoted, and stored in a −80°C freezer within 2 h of collection, in accordance with the TBI Common Data Elements Biospecimens and Biomarkers Working Group Guidelines.^17^ Samples were shipped overnight on dry ice to a central repository and then from the central repository to the University of Pennsylvania for lipid analysis. Non-fasting plasma lipid levels were measured in a single batch from stored frozen plasma using the Roche Cobas 311 instrument at the University of Pennsylvania. Total cholesterol and triglycerides were measured using the enzymatic colorimetric method, and high-density lipoprotein (HDL) cholesterol was measured via direct homogeneous/enzymatic colorimetric methods. Low-density lipoprotein (LDL) cholesterol values were calculated using the Friedewald formula.^18^ The precision and repeatability of the assays were quantified among 21 samples (3 aliquots per run, 1 run per day for 21 days for total cholesterol and triglycerides and 4 aliquots per run, 1 run per day for 21 days for HDL cholesterol) by calculating the coefficient of variation (average of each within-sample ratio of the standard deviation to the mean). The coefficients of variation were excellent for total cholesterol (0.7–1.6%), HDL cholesterol (0.5–2.2%), and triglycerides (0.7–2.0%). Lipid values were analyzed categorically (quartiles) and continuously.

### TBI outcomes

The primary TBI outcome is the GOSE TBI Version,^16,19^ which is a measure of functional impairment resulting from TBI (i.e., not due to other comorbidities or co-occurring polytrauma) that is either self- or proxy-reported. Scores range from 1 (deceased) to 8 (upper good recovery). The GOSE was administered at 2 weeks, 3 months, 6 months, and 1-year post-injury. For analyses, GOSE scores were dichotomized as follows: (1) favorable (GOSE 5–8) versus unfavorable (GOSE 1–4) outcome and (2) complete (GOSE 8) versus incomplete (GOSE 1–7) recovery. In secondary analyses, we restricted the analysis of favorable versus unfavorable outcome to participants with GCS 3–12 (*n* = 135) and the analysis of complete versus incomplete recovery to participants with GCS 13–15 (*n* = 131) to further account for potential confounding by injury severity.

Secondary TBI outcomes include the Rivermead Post-Concussion Symptoms Questionnaire (RPQ),^20^ the Satisfaction with Life Scale (SWLS),^21^ the 18-item Brief Symptom Inventory Global Severity Index (BSI-18-GSI),^22^ and a measure of global cognition.^23^ The RPQ, SWLS, and BSI-18-GSI were administered at 2 weeks, 3 months, 6 months, and 1-year post-injury, and cognitive tests were administered at 2 weeks, 6 months, and 1-year post-injury. The RPQ^20^ is a measure of self-reported injury-related symptoms. The RPQ score range is 0–64, with higher scores indicating more severe symptoms. The SWLS^21^ is a self-reported measure of life satisfaction. The score range is 5–35, with higher scores indicating greater life satisfaction. The BSI-18-GSI^22^ is a self-reported measure of psychological distress. The score range is 0–72, with higher scores indicating greater psychological distress. Cognitive assessments included the Rey Auditory Verbal Learning Test (RAVLT)^24^ immediate and delayed recall (higher scores reflect better verbal episodic memory performance), the Trail Making Test (TMT) Parts A and B^25^ (lower score reflects better executive function performance), and the Wechsler Adult Intelligence Scale—Fourth Edition Processing Speed Index^26^ (higher score reflects better processing speed performance). For analysis, a global cognitive factor score was calculated using confirmatory factor analysis.^23^ One model was fit for each follow-up time point, and correlated residuals were allowed between the two RAVLT items and between the two TMT items. Longitudinal measurement invariance testing demonstrated that the global cognitive factor was reasonably invariant to time. For each participant, calculated global cognitive factor scores from the strict invariance model were used in analyses.

### Statistical analysis

Linear regression models were used to compare group differences (TBI versus controls; TBI severity groups) in lipid levels adjusted for age and sex. *p* Values were adjusted for multiple comparisons using Tukey’s method.

Among participants with TBI, generalized estimating equation (GEE) models with a binomial link function were used to assess the associations of day 1 lipids with GOSE outcomes at 2 weeks, 3 months, 6 months, and 12 months post-injury, adjusting for age, sex, race, education, GCS and head CT, and log-transformed glial fibrillary acidic protein (GFAP) (Supplementary Data). Similar GEE models with a Gaussian link function were used to assess the associations of day 1 lipids with 1-year post-injury trajectories of secondary outcomes (RPQ, SWLS, BSI-18-GSI, and global cognitive factor score). These models were adjusted for age, sex, race, education, GCS and head CT, and log-transformed GFAP. For statistical modeling of associations of lipids with TBI outcomes, each lipid value was standardized using the TBI sample mean and standard deviation.

In sensitivity analyses, we repeated our primary analyses (age- and sex-adjusted lipid levels and associations of day 1 lipids with GOSE outcomes) after excluding 21 individuals with TBI and 3 orthopedic trauma controls who reported using lipid-lowering medications at study enrollment in order to account for the possibility that lower levels of lipids were being driven by lipid-lowering medication use.

R Version 4.4.1 (Vienna, Austria)^27^ was used for all statistical analyses, and a two-sided *p* value <0.05 was considered statistically significant.

## Results

The 266 included participants with TBI were a mean age of 39.6 years, 31.2% were female, 14.3% were of Black race, 19.6% had hypertension, 7.1% had diabetes, 31.4% were smokers, 9.0% had hyperlipidemia, and 7.9% were prescribed lipid-lowering medications (Table 1). Compared with participants with TBI, orthopedic trauma controls were older (mean age = 43.0 years), a greater proportion were female (47.1%), whereas healthy controls were younger (mean age = 33.7 years), and a smaller proportion were female (25.9%). Participants with TBI were more likely than orthopedic controls to have major extracranial injury (24.1% versus 16.2%) and had higher median GFAP levels (1,114 pg/nL versus 10 pg/nL).

**Table 1.** Participant Characteristics

	TBI (*n* = 266)	Orthopedic trauma controls (*n* = 68)	Healthy controls (*n* = 58)
Age (years), mean (SD)	39.6 (16.6)	43.0 (16.6)	33.7 (12.5)
Sex, *n* (%)			
Female	83 (31.2)	32 (47.1)	15 (25.9)
Male	183 (68.8)	36 (52.9)	43 (74.1)
Race, *n* (%)			
Black	38 (14.3)	11 (16.4)	—
White	208 (78.2)	54 (80.6)	—
Other	20 (7.5)	2 (3.0)	—
Ethnicity, *n* (%)			
Hispanic	29 (10.9)	14 (20.9)	—
Non-Hispanic	236 (89.1)	53 (79.1)	—
Education (years), mean (SD)	13.5 (2.7)	14.3 (2.7)	—
Prior TBI, *n* (%)	51 (20.3)	9 (14.3)	—
Psychiatric disease, *n* (%)	74 (27.8)	20 (29.4)	—
Hypertension, *n* (%)	52 (19.6)	16 (24.2)	—
Diabetes, *n* (%)	19 (7.1)	4 (6.1)	—
Smoking, *n* (%)	83 (31.4)	13 (19.1)	—
Hyperlipidemia, *n* (%)	24 (9.0)	3 (4.6)	—
Lipid-lowering medication use, *n* (%)	21 (7.9)	3 (4.4)	—
Total cholesterol (mg/dL), mean (SD)	114.5 (39.6)	139.5 (35.6)	123.9 (32.6)
HDL cholesterol (mg/dL), mean (SD)	38.3 (14.7)	43.6 (14.4)	34.8 (13.1)
LDL cholesterol (mg/dL), mean (SD)	57.8 (30.0)	78.8 (27.6)	68.1 (22.5)
Triglycerides (mg/dL), mean (SD)	91.9 (62.8)	85.8 (58.6)	104.6 (65.3)
GFAP (pg/nL), median (IQR)	1114 (203, 3482)	10 (5, 20)	—
Major extracranial injury, *n* (%)	64 (24.1)	11 (16.2)	—

The following variables contained missing data: ethnicity (TBI, *n* = 1; orthopedic trauma controls *n* = 1), education (orthopedic trauma controls, *n* = 1), prior TBI (TBI, *n* = 14; orthopedic trauma controls, *n* = 5), hypertension (orthopedic trauma controls, *n* = 2), diabetes (orthopedic trauma controls, *n* = 2), smoking (TBI, *n* = 2), and hyperlipidemia (orthopedic trauma controls, *n* = 2). Information on race/ethnicity, education, prior TBI, psychiatric disease, hypertension, diabetes, smoking, hyperlipidemia, lipid-lowering medication use, GFAP, and major extracranial injury were not collected among healthy controls.

GCS, Glasgow Coma Scale; GFAP, glial fibrillary acid protein; HDL, high-density lipoprotein; IQR, interquartile range; LDL, low-density lipoprotein; SD, standard deviation; TBI, traumatic brain injury.

Age- and sex-adjusted total cholesterol and LDL cholesterol were lower among participants with TBI compared with both orthopedic trauma controls and healthy controls (total cholesterol: TBI 116.7 mg/dL [95% confidence interval or CI = 112.1–121.3]; orthopedic trauma controls 137.8 mg/dL [95% CI = 129.1–146.5]; healthy controls 130.2 mg/dL [95% CI = 120.6–139.9] and LDL cholesterol: TBI 59.3 mg/dL [95% CI = 55.8, 62.9]; orthopedic trauma controls 77.7 mg/dL [95% CI = 71.1–84.3]; healthy controls 72.4 mg/dL [95% CI = 65.1–79.7]) (Table 2). Participants with TBI had similar age- and sex-adjusted HDL cholesterol and triglyceride levels to orthopedic injury controls and healthy controls. In sensitivity analyses excluding 24 individuals who reported taking lipid-lowering medications, age- and sex-adjusted lipid levels were similar to our main analysis when comparing individuals with TBI to orthopedic trauma controls (Supplementary Table S1).

**Table 2.** Age- and Sex-Adjusted Lipid Levels (Mean with 95% Confidence Intervals)

	TBI (*n* = 266)	Orthopedic trauma controls (*n* = 68)	Healthy controls (*n* = 58)	*p* Value^*^ TBI versus orthopedic trauma controls	*p* Value^*^ TBI versus healthy controls	*p* Value^*^ orthopedic trauma controls versus healthy controls
Total cholesterol (mg/dL)	116.7 (112.1, 121.3)	137.8 (129.1, 146.5)	130.2 (120.6, 139.9)	<0.001	0.03	0.49
HDL cholesterol (mg/dL)	39.9 (38.2, 41.7)	43.6 (40.3, 46.9)	37.3 (33.7, 41.0)	0.13	0.40	0.04
LDL cholesterol (mg/dL)	59.3 (55.8, 62.9)	77.7 (71.1, 84.3)	72.4 (65.1, 79.7)	<0.001	0.004	0.55
Triglycerides (mg/dL)	87.3 (79.6, 95.1)	82.9 (68.3, 97.6)	102.4 (86.2, 118.5)	0.86	0.21	0.19

^*^ *p* Values adjusted for multiple comparisons using Tukey’s method.

HDL, high-density lipoprotein; LDL, low-density lipoprotein; TBI, traumatic brain injury.

**FIG. 1.**
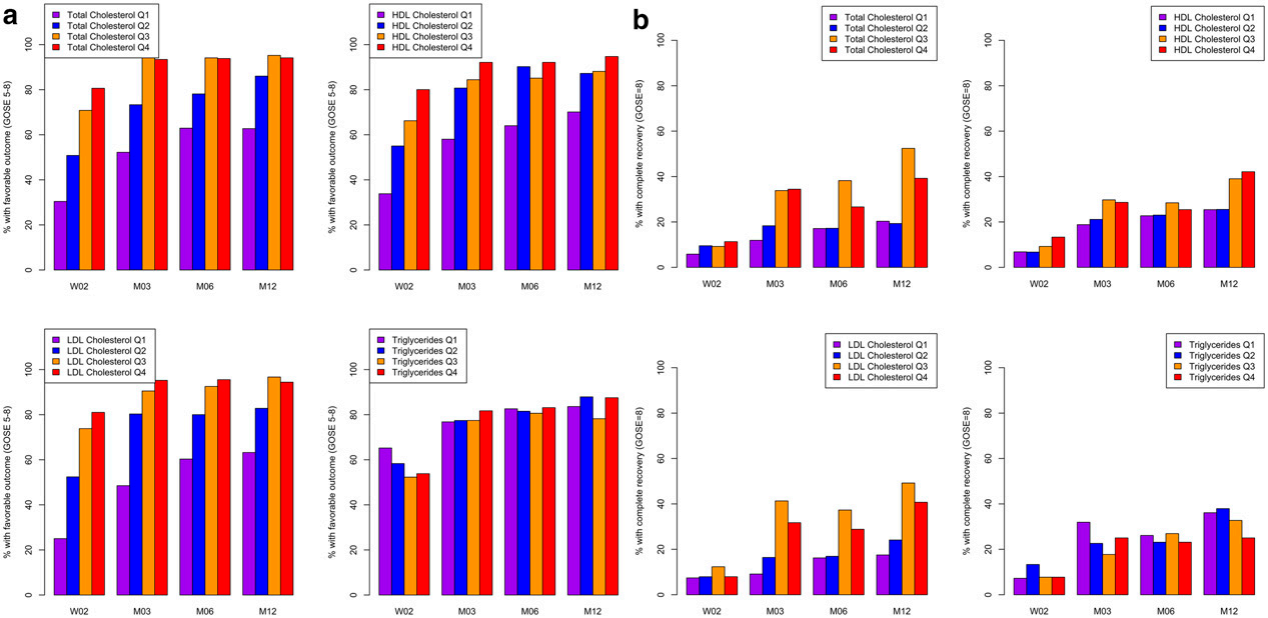
Glasgow Outcome Scale–Extended TBI Version scores (5–8 versus 1–4 and 8 versus 1–7) at 2 weeks, 3 months, 6 months, and 1-year post-injury by quartiles of day 1 post-injury lipids, among individuals with TBI, *n* = 266. Quartiles of total cholesterol: Q1 ≤87 mg/dL, Q2 >87–112 mg/dL, Q3 >112–138 mg/dL, Q4 >138 mg/dL. Quartiles of HDL cholesterol: Q1 ≤29 mg/dL, Q2 >29–36 mg/dL, Q3 >36–46.75 mg/dL, Q4 >46.75 mg/dL. Quartiles of LDL cholesterol: Q1 ≤36 mg/dL, Q2 >36–54.5 mg/dL, Q3 >54.5–74 mg/dL, Q4 >74 mg/dL. Quartiles of triglycerides: Q1 ≤50 mg/dL, Q2 >50–70 mg/dL, Q3 >70–105.75 mg/dL, Q4 = >105.75 mg/dL. HDL, high-density lipoprotein; LDL, low-density lipoprotein; TBI, traumatic brain injury.

Among participants with TBI, higher day 1 post-injury total cholesterol levels were associated with favorable (GOSE score 5–8) versus unfavorable (GOSE score 1–4) outcome at 3 months, 6 months, and 12 months post-injury (Table 3; Fig. 1). Higher day 1 LDL cholesterol was associated with greater odds of favorable outcome at 3 months (odds ratio [OR] = 2.68, 95% CI = 1.51–4.75) and 6 months (OR = 2.21, 95% CI = 1.27–3.85) post-injury, and higher triglycerides cholesterol levels were associated with greater odds of favorable outcome 1-year post-injury (OR = 1.61, 95% CI = 1.02–2.52). There were no statistically significant associations of day 1 post-injury lipids with complete (GOSE score 8) versus incomplete (GOSE score 1–7) recovery (Table 3; Fig. 1). In sensitivity analyses excluding 21 individuals who reported taking lipid-lowering medications, results were similar to the main analyses (Supplementary Table S2).

**Table 3.** Adjusted^a^ Associations of Day 1 Post-Injury Lipids with Glasgow Outcome Scale–Extended Traumatic Brain Injury Version Scores, Among Individuals with Traumatic Brain Injury, *n* = 266

	Total cholesterol OR (95% CI)	HDL OR (95% CI)	LDL OR (95% CI)	Triglycerides OR (95% CI)
Favorable (GOSE 5–8) versus unfavorable (GOSE 1–4) outcome				
2 weeks	1.61 (0.97, 2.67)	1.54 (0.91, 2.59)	1.45 (0.81, 2.58)	0.91 (0.60, 1.37)
3 months	**3.14 (1.60, 6.18)**	1.78 (0.99, 3.20)	**2.68 (1.51, 4.75)**	1.32 (0.88, 1.99)
6 months	**2.33 (1.28, 4.24)**	1.35 (0.80, 2.28)	**2.21 (1.27, 3.85)**	1.28 (0.88, 1.86)
12 months	**2.37 (1.12, 5.02)**	1.54 (0.78, 3.02)	1.82 (0.98, 3.99)	**1.61 (1.02, 2.52)**
Complete (GOSE 8) versus incomplete (GOSE 1–7) recovery				
2 weeks	1.05 (0.63, 1.76)	0.90 (0.61, 1.34)	0.99 (0.60, 1.64)	1.28 (0.88, 1.86)
3 months	1.35 (0.96, 1.91)	1.07 (0.78, 1.47)	1.39 (1.00, 1.94)	1.01 (0.71, 1.43)
6 months	1.01 (0.73, 1.41)	0.82 (0.61, 1.12)	1.06 (0.76, 1.48)	1.10 (0.82, 1.48)
12 months	1.08 (0.77, 1.50)	1.10 (0.80, 1.51)	1.05 (0.76, 1.46)	0.96 (0.67, 1.39)

Bolded values indicate *p* < 0.05.

^a^ GEE models adjusted for age (years, continuous), sex (female; male), race (Black; non-Black), education (years, continuous), GCS/head CT (GCS 3–12; GCS 13–15 and CT positive; GCS 13–15 and CT negative), and log-transformed GFAP.

CI, confidence interval; CT, computed tomography; GCS, Glasgow Coma Scale; GEE, generalized estimating equations; GFAP, glial fibrillary acid protein; GOSE, Glasgow Outcome Scale–Extended; HDL, high-density lipoprotein; LDL, low-density lipoprotein; OR, odds ratio.

The subsets of participants with data on secondary outcomes were similar to the 266 participants included in the primary GOSE analyses in terms of demographics and comorbidities, but participants with cognitive data (*n* = 154) had lower median (interquartile range [IQR]) GFAP (435 pg/nL [66–1346] versus 1114 pg/nL [203–3482]), and participants with RPQ, SWLS, and BSI-18-GSI (*n* = 170) had higher median (IQR) GFAP (1504 pg/nL [452–9629]) (Supplementary Table S3). Higher day 1 post-injury total cholesterol and LDL cholesterol were associated with greater decline in RPQ score over the first-year post-injury (Table 4). Day 1 post-injury lipids were not associated with satisfaction with life (SWLS), psychological distress (BSI-18-GSI), or global cognition over the first-year post-injury.

**Table 4.** Adjusted^a^ Associations of Day 1 Post-Injury Lipids with Traumatic Brain Injury Symptoms, Satisfaction with Life, Psychological Distress, and Cognition, Among Individuals with Traumatic Brain Injury

	Total cholesterol β (95% CI)	HDL β (95% CI)	LDL β (95% CI)	Triglycerides β (95% CI)
Rivermead Post-Concussion Symptoms Questionnaire				
2 weeks	−0.08 (−2.51, 2.36)	0.43 (−1.71, 2.57)	−0.32 (−2.88, 2.25)	−0.02 (−2.11, 2.08)
Change 2 weeks to 6 months	−0.54 (−2.42, 1.34)	−0.12 (−2.17, 1.93)	−0.78 (−2.69, 1.12)	0.35 (−1.24, 1.93)
Change 2 weeks to 12 months	**−1.90 (−3.74, −0.05)**	−1.85 (-4.07, 0.36)	**−1.98 (−3.91, −0.05)**	1.18 (−0.14, 2.50)
Satisfaction with Life Scale				
2 weeks	−0.58 (−1.54, 0.38)	−0.65 (−1.55, 0.26)	−0.16 (−1.19, 0.87)	−0.61 (−1.50, 0.28)
Change 2 weeks to 6 months	0.17 (−0.13, 1.47)	0.29 (−0.83, 1.41)	0.10 (−1.23, 1.42)	−0.02 (−1.03, 0.99)
Change 2 weeks to 12 months	0.13 (−0.96, 1.21)	−0.06 (−1.08, 0.95)	0.37 (−0.77, 1.51)	−0.38 (−1.32, 0.57)
18-Item Brief Symptom Inventory Global Severity Index				
2 weeks	−0.46 (−2.70, 1.77)	1.25 (−0.63, 3.13)	−1.40 (−3.83, 1.03)	0.39 (−1.86, 2.63)
Change 2 weeks to 6 months	0.63 (−0.92, 2.17)	−0.24 (−1.72, 1.24)	0.61 (−0.90, 2.12)	0.78 (−0.79, 2.34)
Change 2 weeks to 12 months	−0.87 (−2.53, 0.79)	−0.96 (−2.77, 0.85)	−0.59 (−2.35, 1.16)	0.01 (−1.26, 1.27)
Global Cognitive Factor Score				
2 weeks	0.05 (−0.07, 0.17)	0.03 (−0.08, 0.14)	0.02 (−0.09, 0.13)	0.06 (−0.07, 0.19)
Change 2 weeks to 6 months	−0.08 (−0.05, 0.04)	0.01 (−0.04, 0.05)	−0.01 (−0.05, 0.04)	−0.01 (−0.06, 0.03)
Change 2 weeks to 12 months	−0.01 (−0.06, 0.03)	0.02 (−0.03, 0.07)	−0.02 (−0.07, 0.02)	−0.02 (−0.07, 0.03)

Bolded values indicate *p* < 0.05.

*n* = 170 with Rivermead Post-Concussion Symptoms Questionnaire, Satisfaction with Life Scale, and 18-Item Brief Symptom Inventory Global Severity Index data. *n* = 154 with global cognitive factor score data.

^a^ GEE models adjusted for age (years, continuous), sex (female; male), race (Black; non-Black), education (years, continuous), GCS/head CT (GCS 3–12; GCS 13–15 and CT positive; GCS 13–15 and CT negative), and log-transformed GFAP.

CT, computed tomography; GCS, Glasgow Coma Scale; GEE, generalized estimating equations; GFAP, glial fibrillary acid protein; HDL, high-density lipoprotein; LDL, low-density lipoprotein.

In secondary analyses among individuals with TBI, 86 had GCS 13–15 with negative head CT, 45 had GCS 13–15 with positive head CT, and 135 had GCS 3–12. Participants with GCS 13–15 with positive head CT were older compared with participants GCS 13–15 with negative head CT and to participants with GCS 3–12 (49.1 years versus 38.7 years versus 37.1 years, respectively) (Table 5). Participants with GCS 13–15 with positive versus negative head CT had similar levels of all lipids (Table 6). Compared to participants with GCS 13–15 (with either positive or negative CT), participants with greater injury severity (GCS 3–12) had significantly lower age- and sex-adjusted levels of total cholesterol, HDL cholesterol, and LDL cholesterol, but similar triglyceride levels. In sensitivity analyses excluding 21 individuals who reported taking lipid-lowering medications, age- and sex-adjusted lipid levels were similar to those estimated in the main TBI severity analysis (Supplementary Table S4). In analyses restricted to individuals with GCS 3–12, higher day 1 total cholesterol was associated with greater adjusted odds of favorable (GOSE score 5–8) versus unfavorable (GOSE score 1–4) outcome at 3 months, 6 months, and 1-year post-injury; higher day 1 HDL cholesterol was associated with greater odds of favorable recovery at 2 weeks and 3 months post-injury; and higher day 1 LDL cholesterol was associated with greater odds of unfavorable recovery at all follow-up time points (Supplementary Table S5). Among participants with GCS 13–15, day 1 lipids were not associated with complete (GOSE score 8) versus incomplete (GOSE score 1–7) recovery.

**Table 5.** Participant Characteristics, Stratified by Traumatic Brain Injury Severity

	TBI GCS 13–15 and CT negative (*n* = 86)	TBI GCS 13–15 and CT positive (*n* = 45)	TBI GCS 3–12 (*n* = 135)
Age (years), mean (SD)	38.7 (15.2)	49.1 (19.5)	37.1 (15.3)
Sex, *n* (%)			
Female	34 (39.5)	18 (40.0)	31 (23.0)
Male	52 (60.5)	27 (60.0)	104 (77.0)
Race, *n* (%)			
Black	19 (22.1)	1 (2.2)	18 (13.3)
White	59 (68.6)	42 (93.3)	107 (79.3)
Other	8 (9.3)	2 (4.4)	10 (7.4)
Ethnicity, *n* (%)			
Hispanic	7 (8.1)	9 (20.0)	13 (9.7)
Non-Hispanic	79 (91.9)	36 (80.0)	121 (90.3)
Education (years), mean (SD)	14.1 (2.7)	14.5 (3.2)	12.7 (2.3)
Prior TBI, *n* (%)	28 (34.6)	6 (14.0)	17 (13.4)
Psychiatric disease, *n* (%)	30 (34.9)	11 (24.4)	33 (24.4)
Hypertension, *n* (%)	18 (20.9)	11 (24.4)	23 (17.0)
Diabetes, *n* (%)	4 (4.7)	7 (15.6)	8 (5.9)
Smoking, *n* (%)	26 (30.6)	8 (17.8)	49 (36.6)
Hyperlipidemia, *n* (%)	9 (10.5)	6 (13.3)	9 (6.7)
Lipid-lowering medication use, *n* (%)	9 (10.5)	5 (11.1)	7 (5.2)
Total cholesterol (mg/dL), mean (SD)	135.7 (38.9)	127.2 (32.4)	98.8 (33.8)
HDL cholesterol (mg/dL), mean (SD)	42.7 (14.5)	44.4 (14.6)	33.4 (13.3)
LDL cholesterol (mg/dL), mean (SD)	74.6 (30.5)	66.7 (27.7)	44.2 (23.3)
Triglycerides (mg/dL), mean (SD)	91.7 (65.2)	80.0 (44.0)	95.9 (66.3)
GFAP (pg/nL), median (IQR)	124 (22, 456)	1047 (348, 1973)	2925 (1194, 6280)
Major extracranial injury, *n* (%)	15 (17.4)	8 (17.8)	41 (30.4)

The following variables contained missing data: ethnicity (*n* = 1), prior TBI (*n* = 14), and smoking (*n* = 2).

CT, computed tomography; GCS, Glasgow Coma Scale; GFAP, glial fibrillary acid protein; HDL, high-density lipoprotein; IQR, interquartile range; LDL, low-density lipoprotein; SD, standard deviation; TBI, traumatic brain injury.

**Table 6.** Age- and Sex-Adjusted Lipid Levels (Mean with 95% Confidence Intervals), Stratified by Traumatic Brain Injury Severity

	TBI GCS 13–15 and CT negative (*n* = 86)	TBI GCS 13–15 and CT positive (*n* = 45)	TBI GCS 3–12 (*n* = 135)	*p* Value^*^ TBI GCS^13–15^ and CT negative versus positive	*p* Value^*^ TBI GCS^13–15^ and CT negative versus GCS 3–12	*p* Value^*^ TBI GCS^13–15^ and CT positive versus GCS ^3–12^
Total cholesterol (mg/dL)	136.3 (128.9, 143.8)	123.3 (112.7, 133.8)	98.6 (92.2, 105.0)	0.11	<0.001	<0.001
HDL cholesterol (mg/dL)	43.3 (40.4, 46.3)	45.1 (41.0, 49.3)	35.0 (32.4, 37.5)	0.76	<0.001	<0.001
LDL cholesterol (mg/dL)	75.0 (69.4, 80.6)	64.0 (56.0, 72.0)	45.4 (40.6, 50.3)	0.07	<0.001	<0.001
Triglycerides (mg/dL)	89.8 (76.7, 102.8)	70.6 (52.1, 89.1)	91.0 (79.7, 102.3)	0.22	0.99	0.15

^*^ *p* Values adjusted for multiple comparisons using Tukey’s method.

CT, computed tomography; GCS, Glasgow Coma Scale; HDL, high-density lipoprotein; LDL, low-density lipoprotein; TBI, traumatic brain injury.

## Discussion

In this prospective study of individuals with acute TBI, we found that day 1 post-injury levels of total and LDL cholesterol were lower among individuals with TBI compared with both orthopedic trauma and healthy controls. This finding held among individuals with more severe versus less severe TBI. We further found that individuals who did not have acutely low total cholesterol and LDL cholesterol, and to a lesser extent triglycerides, had better functional outcomes over all time points in the first-year post-TBI. Taken together, these findings suggest that lower levels of circulating lipids in the acute post-TBI setting may represent biological processes associated with impaired long-term recovery, a component of injury severity not fully captured by other traditional indicators of injury severity such as GCS, head CT findings, and GFAP, or a combination thereof.

Consistent with prior work in the broader trauma literature^5–7^ and within the TBI field,^10,11,13^ we observed lower levels of total and LDL cholesterol among participants with TBI compared with healthy controls. In contrast to these prior studies,^5–7^ we also observed lower levels of total and LDL cholesterol among participants with TBI compared with orthopedic trauma controls. These results may in part be explained by the smaller proportion of individuals with major extracranial injury in the orthopedic control group compared with the TBI group. We also observed lower levels of total, HDL, and LDL cholesterol among participants with TBI of greater versus lesser severity, as assessed by the GCS and head CT. Importantly, in our study, we compared age- and sex-adjusted lipid levels by participant group given the notable age and sex differences between certain groups and between certain TBI severity categories; however, there remains the possibility of residual confounding by these characteristics.

Our work expands the evidence base by examining associations of acutely measured lipids with long-term clinically relevant outcomes after TBI. Prior work in TBI has largely focused on associations of lipids measured in the acute setting post-injury with other biomarker outcomes, such as inflammatory cytokines,^10^ hemorrhage expansion,^13^ or diffuse axonal injury on neuroimaging,^11^ although some have also looked at clinical outcomes in individuals with greater injury severity, such as the recovery of consciousness within 6 months after severe TBI.^11^ It is interesting to note that while the literature is generally consistent regarding lower lipid levels being associated with adverse outcomes,^5–7,10,11,13^ it is mixed regarding which specific lipids (total cholesterol, LDL cholesterol, HDL cholesterol, triglycerides) are associated. For example, Zhong et al.^11^ found that individuals with lower HDL cholesterol were less likely to regain consciousness within 6 months post-injury. Kim et al.^13^ reported that lower triglycerides, but not total cholesterol, LDL cholesterol, or HDL cholesterol, were associated with hemorrhage expansion, while Venetsanou et al.^10^ reported that LDL cholesterol levels were lower among TBI non-survivors compared to survivors. Our study suggests that acutely measured total cholesterol and LDL cholesterol, and to a lesser extent triglycerides, are associated with functional outcomes over the first-year post-injury. There is a clear need to better understand the patterns and potential implications of lipid profiles in the acute post-TBI time period.

The results of this study should be interpreted in the context of certain limitations. First, this analysis was performed among a subset of TRACK-TBI Study participants, and results may not be representative of the full cohort, although the analytic subset included individuals with injuries of all severities. Furthermore, our study may have been underpowered to detect associations, particularly for secondary and stratified analyses. Second, lipids were measured at one time point post-injury, which precluded investigation of associations of the change in lipid values with outcomes. Furthermore, we did not have pre-injury lipid measurements. Therefore, we were unable to discriminate between pre-existing hypocholesterolemia versus acute trauma-induced hypocholesterolemia in our study. However, the results of our sensitivity analyses excluding the minority of participants who reported use of lipid-lowering medications were similar to our main analyses, which suggests that our results are more driven by acute trauma-induced hypocholesterolemia, though data on lipid-lowering medication use were not collected among healthy controls in our study. Future studies should consider the inclusion of both pre- and post-injury lipid measurements in addition to detailed medication histories. Given the small number of participants who reported having a pre-injury diagnosis of hyperlipidemia or being prescribed lipid-lowering medications, we were unable to fully investigate the potential impact of chronic hyperlipidemia or statin use on the observed associations. Investigation of associations of lipid values, with consideration of the impact of lipid-lowering medication use, on TBI outcomes deserves further study given the prior literature, which has suggested neuroprotective effects of statins in TBI.^28–30^

In conclusion, our study suggests that TBI, and in particular, more severe TBI (GCS 3–12) is associated with lower levels of circulating lipids, namely total and LDL cholesterol, in the acute post-injury time period. Furthermore, individuals with TBI who did not have acutely low lipid levels were more likely to have better functional outcomes over the first-year post-injury. Further work is needed to better understand the mechanisms underlying hypocholesterolemia in acute TBI and to more fully understand the clinical implications of hypocholesterolemia in the acute TBI setting.

## Transparency, Rigor, and Reproducibility Summary

The TRACK-TBI Study was pre-registered at clinicaltrials.gov (clinicaltrials.gov NCT2119182). The TRACK-TBI clinical protocol and outcome procedures are available at https://tracktbi.ucsf.edu/researchers. All data used for this investigation are available by request from Federal Interagency Traumatic Brain Injury Research (https://fitbir.nih.gov/). All codes and scripts used for this investigation are available from the corresponding author upon reasonable request.

## Abbreviations Used

BSI-18-GSI: 8-item Brief Symptom Inventory Global Severity Index
CT: Computed Tomography
GCS: Glasgow Coma Scale
GEE: Generalized Estimating Equation
GFAP: Glial Fibrillary Acid Protein
GOSE: Glasgow Outcome Scale–Extended
HDL: High-density Lipoprotein
IRB: Institutional Review Board
LDL: Low-density Lipoprotein
RAVLT: Rey Auditory Verbal Learning Test
RPQ: Rivermead Post Concussion Symptoms Questionnaire
SWLS: Satisfaction with Life Scale
TBI: Traumatic Brain Injury
TMT: Trail Making Test
TRACK-TBI: Transforming Research and Clinical Knowledge in Traumatic Brain Injury
WAIS-IV PSI: Wechsler Adult Intelligence Scale—Fourth Edition Processing Speed Index
