# Supplementary material for: Associations of Plasma Lipids with Traumatic Brain Injury Outcomes: A Transforming Research and Clinical Knowledge in Traumatic Brain Injury Study
Source: Neurotrauma Rep. 2025 Nov 11;6(1):1014–23. doi: 10.1177/2689288X251395462 (PMC12755847; doi:10.1177/2689288X251395462)
Supplement: Supplementary Data [file 2689288x251395462_supplementary_data.docx]

**Online Only Supplemental Materials**

Schneider ALC, Brett BL, Abbruzzese S, Sandsmark DK, Jain S, Sun X, Gardner RC, Manley GT, Nelson LD, Diaz-Arrastia R, TRACK-TBI Study Investigators. Associations of Plasma Lipids with Traumatic Brain Injury Outcomes: A TRACK-TBI Study

**Table of Contents**

[eMethods. Covariates and Other Variables. 2](#_Toc210745382)

[eTable 1. Age- and Sex-Adjusted Lipid Levels (Mean with 95% Confidence Intervals), Excluding 24 Individuals Taking Lipid-lowering Medications. 3](#_Toc210745383)

[eTable 2. Adjusted* Associations of Day 1 Post-Injury Lipids with Glasgow Outcome Scale-Extended TBI Version Scores, Among Individuals with TBI, Excluding 21 Individuals Taking Lipid-lowering Medications. 4](#_Toc210745384)

[eTable 3. Participant Characteristics, Stratified by Availability of Outcome Data. 5](#_Toc210745385)

[eTable 4. Age- and Sex-Adjusted Lipid Levels (Mean with 95% Confidence Intervals), Stratified by TBI Severity, Excluding 21 Individuals Taking Lipid-lowering Medications. 6](#_Toc210745386)

[eTable 5. Adjusted* Associations of Day 1 Post-Injury Lipids with Glasgow Outcome Scale-Extended TBI Version Scores, Among Individuals with TBI, Stratified by Injury Severity. 7](#_Toc210745387)

[eReferences. 8](#_Toc210745388)

# eMethods. Covariates and Other Variables.

The following covariates (confounders) were included in statistical models: age (years; continuous), sex (female; male), race (Black; non-Black), education (years; continuous), and 2 indices of TBI injury severity: 1) GCS and head CT findings: GCS 13-15 and CT negative; GCS 13-15 and CT positive; GCS 3-12 (head CT positive versus negative for intracranial trauma-related abnormalities was defined according to the TBI Common Data Elements Neuroimaging Working Group expert consensus recommendations),^1, 2^ and 2) log transformed day 1 glial fibrillary acid protein (GFAP).

Other variables include: hypertension, diabetes, smoking, and hyperlipidemia (yes; no, defined from self- or proxy-report, medical record review, and self- or proxy-reported medication use, as previously described^3^), psychiatric disease (yes; no, self- or proxy-reported), lipid-lowering medication use (yes; no, defined by self- or proxy-reported use of statins, colesevelam, ezetimibe, fenofibrate, gemfibrozil^3^), and presence of major extracranial injuries (yes; no, defined as an Abbreviated Injury Scale score calculated for regions below the neck of ≥3). As the Abbreviated Injury Scale is only available for hospitalized patients, individuals who were not hospitalized were assumed to have no major extracranial injury, in accordance with prior studies.^4^

# eTable 1. Age- and Sex-Adjusted Lipid Levels (Mean with 95% Confidence Intervals), Excluding 24 Individuals Taking Lipid-lowering Medications.

|  | TBI  (n=345) | Orthopedic  Trauma Controls  (n=65) | P-Value* TBI Versus Orthopedic Trauma Controls |
| --- | --- | --- | --- |
| Total cholesterol (mg/dl) | 117.3  (112.3, 122.3) | 138.3  (129.2, 147.5) | <0.001 |
| HDL cholesterol (mg/dl) | 40.2  (38.2, 42.0) | 43.6  (40.2, 47.0) | 0.08 |
| LDL cholesterol (mg/dl) | 59.8  (56.0, 63.7) | 78.1  (71.1, 85.2) | <0.001 |
| Triglycerides (mg/dl) | 86.2  (78.0, 94.5) | 83.0  (67.9, 98.1) | 0.72 |

*P-Values adjusted for multiple comparisons using Tukey’s Method.

Abbreviations: HDL, high density lipoprotein; LDL, low density lipoprotein; TBI, traumatic brain injury.

# eTable 2. Adjusted* Associations of Day 1 Post-Injury Lipids with Glasgow Outcome Scale-Extended TBI Version Scores, Among Individuals with TBI, Excluding 21 Individuals Taking Lipid-lowering Medications.

|  | Total Cholesterol  OR (95% CI) | HDL  OR (95% CI) | LDL  OR (95% CI) | Triglycerides  OR (95% CI) |
| --- | --- | --- | --- | --- |
| Favorable (GOSE 5-8) Versus Unfavorable (GOSE 1-4) Outcome |  |  |  |  |
| 2-weeks | 1.42 (0.82, 2.45) | 1.47 (0.77, 2.78) | 1.31 (0.72, 2.37) | 1.21 (0.82, 1.79) |
| 3-months | **4.06 (2.16, 7.64)** | **2.33 (1.39, 3.90)** | **2.93 (1.64, 5.24)** | 1.05 (0.75, 1.47) |
| 6-months | **3.01 (1.73, 5.23)** | **1.80 (1.10, 2.93)** | **2.63 (1.48, 4.68)** | 1.09 (0.81, 1.47) |
| 12-months | **3.42 (1.65, 7.08)** | **2.39 (1.37, 4.17)** | **2.22 (1.13, 4.36)** | 1.02 (0.71, 1.46) |
| Complete (GOSE 8) Versus Incomplete (GOSE 1-7) Recovery |  |  |  |  |
| 2-weeks | 0.96 (0.56, 1.64) | 0.90 (0.58, 1.39) | 0.90 (0.52, 1.54) | 1.21 (0.82, 1.79) |
| 3-months | 1.38 (0.95 1.99) | 1.07 (0.76, 1.51) | 1.40 (0.99, 1.97) | 1.05 (0.75, 1.47) |
| 6-months | 0.94 (0.66, 1.35) | 0.81 (0.58, 1.13) | 0.97 (0.68, 1.39) | 1.09 (0.81, 1.47) |
| 12-months | 1.06 (0.74, 1.52) | 1.13 (0.80, 1.60) | 1.00 (0.70, 1.41) | 1.02 (0.71, 1.46) |

*GEE models adjusted for age (years, continuous), sex (female; male), race (Black; non-Black), education (years, continuous), GCS/head CT (GCS 3-12; GCS 13-15 and CT positive; GCS 13-15 and CT negative), and log transformed GFAP.

Bolded values indicate p<0.05.

Abbreviations: GEE, generalized estimating equations; GFAP, glial fibrillary acid protein; HDL, high density lipoprotein; LDL, low density lipoprotein.

# eTable 3. Participant Characteristics, Stratified by Availability of Outcome Data.

|  | TBI + GOSE  (n=266) | TBI + RPQ, SWLS, and BSI-18-GSI  (n=170) | TBI + Global Cognitive Factor Score  (n=154) |
| --- | --- | --- | --- |
| Age (years), mean (SD) | 39.6 (16.6) | 40.1 (16.9) | 39.1 (16.3) |
| Sex, n (%) |  |  |  |
| Female | 83 (31.2) | 63 (37.1) | 56 (36.4) |
| Male | 183 (68.8) | 107 (62.9) | 98 (63.6) |
| Race, n (%) |  |  |  |
| Black | 38 (14.3) | 27 (15.9) | 24 (15.6) |
| White | 208 (78.2) | 132 (77.7) | 119 (77.3) |
| Other | 20 (7.5) | 11 (6.5) | 11 (7.1) |
| Ethnicity, n (%) |  |  |  |
| Hispanic | 29 (10.9) | 16 (9.4) | 15 (9.7) |
| Non-Hispanic | 236 (89.1) | 154 (90.6) | 139 (90.3) |
| Education (years), mean (SD) | 13.5 (2.7) | 14.0 (2.8) | 14.1 (2.7) |
| Prior TBI, n (%) | 51 (20.3) | 40 (24.8) | 37 (25.3) |
| Psychiatric disease, n (%) | 74 (27.8) | 51 (30.0) | 48 (31.2) |
| Hypertension, n (%) | 52 (19.6) | 31 (18.2) | 28 (18.2) |
| Diabetes, n (%) | 19 (7.1) | 13 (7.7) | 11 (7.1) |
| Smoking, n (%) | 83 (31.4) | 47 (27.8) | 43 (28.1) |
| Hyperlipidemia, n (%) | 24 (9.0) | 15 (8.8) | 14 (9.1) |
| Lipid-lowering medication use, n (%) | 21 (7.9) | 13 (7.7) | 12 (7.8) |
| Total cholesterol (mg/dl), mean (SD) | 114.5 (39.6) | 126.5 (37.6) | 129.1 (36.8) |
| HDL cholesterol (mg/dl), mean (SD) | 38.3 (14.7) | 41.5 (14.2) | 41.9 (14.2) |
| LDL cholesterol (mg/dl), mean (SD) | 57.8 (30.0) | 67.8 (28.9) | 69.7 (28.5) |
| Triglycerides (mg/dl), mean (SD) | 91.9 (62.8) | 86.3 (60.0) | 87.1 (60.5) |
| GFAP (pg/nl), median (IQR) | 1114 (203, 3482) | 1504 (452, 9629) | 435 (66, 1346) |

Abbreviations: GCS, Glasgow Coma Scale; GFAP, glial fibrillary acid protein; HDL, high density lipoprotein; IQR, interquartile range; LDL, low density lipoprotein; SD, standard deviation; TBI, traumatic brain injury.

The following variables contained missing data: ethnicity (TBI+GOSE, n=1), prior TBI (TBI+ GOSE, n=14; TBI + RPQ, SWLS, and BSI-18-GSI, n=9; TBI + Global Cognitive Factor Score, n=8), smoking (TBI+GOSE, n=2; TBI + RPQ, SWLS, and BSI-18-GSI, n=1; TBI + Global Cognitive Factor Score, n=1).

# eTable 4. Age- and Sex-Adjusted Lipid Levels (Mean with 95% Confidence Intervals), Stratified by TBI Severity, Excluding 21 Individuals Taking Lipid-lowering Medications.

|  | TBI GCS 13-15 and CT negative (n=77) | TBI GCS 13-15 and CT positive (n=40) | TBI  GCS 3-12  (n=128) | P-Value* TBI GCS 13-15 and CT Negative Versus Positive | P-Value* TBI GCS 13-15 and CT Negative Versus GCS 3-12 | P-Value* TBI GCS 13-15 and CT Positive Versus GCS 3-12 |
| --- | --- | --- | --- | --- | --- | --- |
| Total cholesterol (mg/dl) | 138.2  (130.5, 145.9) | 124.4  (113.4, 135.3) | 98.4  (91.9, 104.9) | 0.11 | <0.001 | <0.001 |
| HDL cholesterol (mg/dl) | 44.3  (41.3, 47.3) | 46.2  (41.9, 50.4) | 34.8  (32.3, 37.3) | 0.77 | <0.001 | <0.001 |
| LDL cholesterol (mg/dl) | 76.4  (70.5, 82.3) | 64.8  (56.4, 73.2) | 45.7  (40.8, 50.6) | 0.07 | <0.001 | <0.001 |
| Triglycerides (mg/dl) | 87.2  (73.1, 101.2) | 67.1  (47.2, 87.1) | 89.7  (78.0, 101.5) | 0.24 | 0.96 | 0.13 |

*P-Values adjusted for multiple comparisons using Tukey’s Method.

Abbreviations: HDL, high density lipoprotein; LDL, low density lipoprotein; TBI, traumatic brain injury.

# eTable 5. Adjusted* Associations of Day 1 Post-Injury Lipids with Glasgow Outcome Scale-Extended TBI Version Scores, Among Individuals with TBI, Stratified by Injury Severity.

|  | Total Cholesterol  OR (95% CI) | HDL cholesterol  OR (95% CI) | LDL cholesterol  OR (95% CI) | Triglycerides  OR (95% CI) |
| --- | --- | --- | --- | --- |
| Favorable (GOSE 5-8) Versus Unfavorable (GOSE 1-4) Outcome Among Individuals with TBI and GCS 3-12 (n=135) |  |  |  |  |
| 2-weeks | 5.32 (0.77, 36.81) | **2.20 (1.26, 3.83)** | **2.25 (1.21, 4.19)** | 0.71 (0.38, 1.35) |
| 3-months | **6.54 (2.47, 17.30)** | **1.84 (1.04, 3.25)** | **2.89 (1.54, 5.43)** | 1.22 (0.80, 1.84) |
| 6-months | **5.21 (2.05, 13.25)** | 1.52 (0.90, 2.54) | **2.53 (1.35, 4.74)** | 1.18 (0.79, 1.76) |
| 12-months | **4.59 (1.75, 12.15)** | 1.69 (0.84, 3.38) | **2.09 (1.05, 4.13)** | 1.32 (0.83, 2.10) |
| Complete (GOSE 8) Versus Incomplete (GOSE 1-7) Recovery Among Individuals with TBI and GCS 13-15 (n=131) |  |  |  |  |
| 2-weeks | 1.15 (0.63, 2.10) | 0.80 (0.50, 1.28) | 1.06 (0.61, 1.87) | 1.54 (0.98, 2.41) |
| 3-months | 1.12 (0.73, 1.70) | 0.90 (0.60, 1.37) | 1.19 (0.80, 1.75) | 1.02 (0.69, 1.50) |
| 6-months | 1.12 (0.72, 1.74) | 0.84 (0.56, 1.25) | 1.22 (0.82, 1.81) | 1.06 (0.70, 1.61) |
| 12-months | 0.84 (0.55, 1.28) | 1.09 (0.73, 1.64) | 0.92 (0.62, 1.35) | 0.68 (0.38, 1.20) |

*GEE models adjusted for age (years, continuous), sex (female; male), race (Black; non-Black), education (years, continuous), and log transformed GFAP.

Abbreviations: GCS, Glasgow Coma Scale; GEE, generalized estimating equations; GFAP, glial fibrillary acid protein; HDL, high density lipoprotein; LDL, low density lipoprotein.

# eReferences.

1. Duhaime, A.C., Gean, A.D., Haacke, E.M., Hicks, R., Wintermark, M., Mukherjee, P., Brody, D., Latour, L., Riedy, G. and Common Data Elements Neuroimaging Working Group Members, P.W.G.M. (2010). Common data elements in radiologic imaging of traumatic brain injury. Arch Phys Med Rehabil 91, 1661-1666.

2. Haacke, E.M., Duhaime, A.C., Gean, A.D., Riedy, G., Wintermark, M., Mukherjee, P., Brody, D.L., DeGraba, T., Duncan, T.D., Elovic, E., Hurley, R., Latour, L., Smirniotopoulos, J.G. and Smith, D.H. (2010). Common data elements in radiologic imaging of traumatic brain injury. J Magn Reson Imaging 32, 516-543.

3. Schneider, A.L.C., Barber, J., Temkin, N., Gardner, R.C., Manley, G., Diaz-Arrastia, R. and Sandsmark, D. (2023). Associations of Preexisting Vascular Risk Factors With Outcomes After Traumatic Brain Injury: A TRACK-TBI Study. J Head Trauma Rehabil 38, E88-E98.

4. Temkin, N., Barber, J., Machamer, J., Sugar, G., Morrissey, M.R., Boase, K., Zahniser, E., Bodien, Y.G., Giacino, J.T., McCrea, M.A., Nelson, L.D., Stein, M.B., Taylor, S., Robertson, C., Okonkwo, D., Manley, G., Dikmen, S. and Investigators, T.-T. (2025). Contribution of Extracranial Injuries to GOSE Scores after Traumatic Brain Injury TBI: A TRACK-Traumatic Brain Injury Study. J Neurotrauma.
